# Translation and cross-cultural adaptation of the ***Clinical Competence Questionnaire*** for use in Brazil**^1^**

**DOI:** 10.1590/1518-8345.1757.2898

**Published:** 2017-06-05

**Authors:** Danielle Ritter Kwiatkoski, Maria de Fátima Mantovani, Evani Marques Pereira, Carina Bortolato-Major, Ângela Taís Mattei, Aida Maris Peres

**Affiliations:** 2 MSc, RN, Hospital Universitário Regional dos Campos Gerais, Secretaria de Estado de Saúde do Paraná, Ponta Grossa, PR, Brazil.; 3 PhD, Associate Professor, Universidade Federal do Paraná, Curitiba, PR, Brazil.; 4 PhD, Associate Professor, Departamento de Enfermagem, Universidade Estadual do Centro-Oeste, Guarapuava, PR, Brazil.; 5 Doctoral student, Universidade Federal do Paraná, Curitiba, PR, Brazil. Professor, Departamento de Enfermagem, Universidade Estadual do Norte do Paraná, Bandeirantes, PR, Brazil. Scholarship holder at Coordenação de Aperfeiçoamento de Pessoal de Nível Superior (CAPES), Brazil.; 6 Doctoral student, Universidade Federal do Paraná, Curitiba, PR, Brazil. Scholarship holder at Coordenação de Aperfeiçoamento de Pessoal de Nível Superior (CAPES), Brazil.

**Keywords:** Clinical Competence, Professional Competence, Teaching, Education Measurement, Validation Studies, Nursing

## Abstract

**Objective:**

translating and transculturally adapting the *Clinical Competence Questionnaire* to Brazilian senior undergraduate Nursing students, as well as measuring psychometric properties of the questionnaire.

**Method:**

a methodological study carried out in six steps: translation of the *Clinical Competence Questionnaire* instrument, consensus of the translations, back-translation, analysis by an expert committee, pre-testing and then presentation of the cross-cultural adaptation process to the developers. Psychometric properties were measured using Cronbach's alpha, intraclass correlation coefficient and content validity index.

**Results:**

the instrument was translated, transculturally adapted and its final version consisted of 48 items. Cronbach's alpha coefficient was 0.90, and the agreement index of the items was 99% for students and 98% for evaluators.

**Conclusion:**

the *Clinical Competence Questionnaire* was translated and adapted to Brazilian students, and the psychometric properties of the Portuguese version of the questionnaire presented satisfactory internal consistency regarding the studied sample.

## Introduction

In the international scenario of nursing education, it is currently a requirement that students master the skills necessary for their training. To ensure the development of knowledge, skills and attitudes for professional practice, it is imperative that an evaluation method be implemented before graduation. 

An accurate, reliable and valid evaluation allows for verifying clinical performance and their preparation/readiness for professional exercise. In general, assessments that rely on knowledge-based tests reflect the effectiveness of teaching, but fail to demonstrate how students could apply knowledge in clinical situations^1^.

Thus, education has evolved from teaching the profession based on tested experience to teaching it based on scientific evidence, and currently the assessment of nursing students should be able to gauge whether the desired learning outcomes are achieved, and whether the competencies of the course were achieved in order to ensure safe and competent care. Although there are innovative methods in nursing that guarantee an evaluation of clinical skills' learning, many others lack scientific evidence or present gaps^2^. 

On the one hand, nursing education in several parts of the world leads a dynamic, critical and reflective profile, involving complex clinical knowledge and the graduate's ability to act in the face of the unexpected. On the other hand, however, it focuses on assessments aimed toward psychomotor skills, to the detriment of a multidimensional assessment that encompasses attributes of clinical competence^3^.

Thus, assessing the clinical competence of nursing students/graduates has demonstrated problems of reliability, subjectivity, validity and bias in their processes^3^ ^-^ ^4^, which prevents them from achieving their real objectives, noting that this evaluation must also converge to knowledge, skills and attitudes, which can be misconceived when evaluating one or two isolated elements^1^ ^,^ ^5^.

The *Clinical Competence Questionnaire* (CCQ) was constructed and validated in Taiwan in 2013 in order to measure the perception of undergraduate nursing students' clinical competence. The CCQ construction was based on the model "From Novice to Expert", by Patrícia Benner, which ranks nurses in six levels of competence: novice, beginner, advanced, competent, proficient and expert^5^.

The CCQ assesses the skills of nursing students acquired in their training. Usually, new graduates enter the job market as a novice nurse, ideally having the possibility of a rapid progression in their career until reaching a competent level. The questionnaire is composed of 47 items, divided into domains that converge to the required competencies of the bachelor's degree in nursing, including professional behaviors, specific skills, general performance, and advanced skills; evaluating aspects such as safe care, professional ethics, clinical thinking, collaboration and communication, basic nursing routines, and technical skills^5^.

This questionnaire was applied to 340 students of the Registered Nursing Program for a Bachelor's degree in nursing sciences. Considering instrument validity, CCQ had its content validated through an expert panel and its construction through factorial analysis, resulting in two factors - nursing behaviors and skills. Reliability was assured by 0.98^5^ ^)^ Cronbach's alpha. 

Considering that evaluation of clinical competencies in nursing practice is an important tool to guide teachers, field supervisors and to verify students' evolution, and considering the need for instruments that are valid and easy to apply, this study aimed to transculturally translate and adapt the CCQ to Brazilian undergraduate nursing students, as well as to measure the questionnaire's psychometric properties. 

## Method 

This is a methodological development study involving the translation, cross-cultural adaptation and obtaining initial psychometric properties of the CCQ instrument, carried out between May 2015 and April 2016. This instrument is divided into two parts. The first one contains 16 items related to nursing professional behaviors, and the second includes 31 items that correspond to professional skills. The instrument is self-contained and based on a five-point Likert scale, which ranges from "do not have a clue" (point 1) and "know in theory, competent in practice without any supervision" (point 5). The respondents were able to indicate how much they agreed with the statements, and their scores obtained by totaling the sum of the item scores varied between 47 and 235. The higher the score, the higher the level of competence^5^.

**Figure 1:**
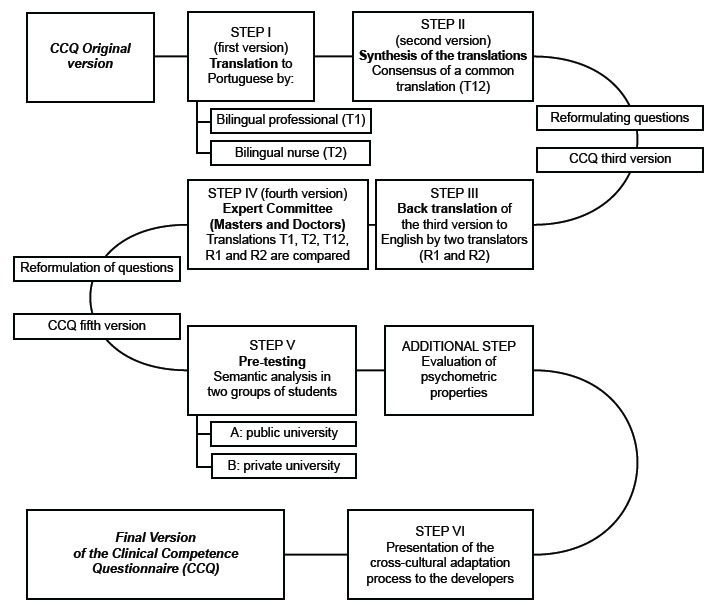
Methodological trajectory of the Clinical Competence Questionnaire cultural adaptation. Curitiba, PR, Brazil, 2016

The CCQ translation process was guided by the theoretical framework of cross-cultural adaptation, and comprised the following steps (Figure 1): translation, synthesis, back-translation, review by the Expert Committee, pre-test measuring psychometric properties and lastly presenting study reports to the developers of the cross-cultural adaptation process^6^.

Two bilingual translators (English/Portuguese) participated in step I. The former had no knowledge of the health sciences area, and the second was a nurse. Two other bilingual translators (English/Portuguese) were invited for step III who had not participated in step I. The expert committee referred to in step IV was comprised of seven masters or doctors, all with experience in the theme and fluent in the English language. In step V, the instrument was applied to 43 nursing graduate students, as recommended by the reference used in this study^6^. 

Statistical analyzes of the pre-test were performed using the Statistical Package for the Social Sciences, version 23. Cronbach's alpha coefficient was used for the reliability analysis, establishing a minimum value of 0.70 to demonstrate that the items would measure the same construct^7^.

In order to assess the instrument's stability, the Intraclass Correlation Coefficient (ICC) was measured in order to guarantee its reproducibility. The following criteria were adopted: values between 0 (zero) and 0.20 = poor; between 0.21 and 0.40 = reasonable; between 0.41 and 0.60 = good; between 0.61 and 0.80 = very good; between 0.81 and 1.00 = excellent^8^.

The relevance and representativeness of the items were evaluated through the Content Validity Index (CVI), which measures agreement among the evaluators. The adequacy of each item ranged from adequate to unsuitable in a Likert scale. A minimum value of 0.90, or 90% was considered^9^.

Meetings were recorded and later transcribed in full for steps I, II, III and IV, comparing the results and adjusting them according to the suggestions of the evaluated items. Reports for each translation, back translation, consensus version of the phases and from the expert committee were carried out. The pre-test was carried out in step V using the version obtained from previous steps.

The use of the CCQ was authorized by the main author of the questionnaire, and the research was approved by the Research Ethics Committee of the Federal University of Paraná, under the opinion 031754/2015. All study participants signed the clear and informed consent form.

## Results

The final instrument totaled 48 items, since item 40 in the Skills and Abilities domain unfolded into items 40 and 41 after the experts suggested that it would be appropriate for "oxygen therapy" to be separated from "performing postural drainage and percussion." Thus, the total score went from 235 to 240.

Regarding content validation, six items - 4, 6, 21, 30, 40 and 41 - were modified, respecting the semantic and cultural equivalences. The remaining 41 items had 98% agreement among the specialists. The consensus among the specialists dictated that all the items were modified using first person singular.

The final version, named *Questionário de Competência Clínica* (QCC), was answered by 43 senior students from two nursing courses. The students assessed themselves as clinically competent, where 238 was the highest score and 202 was the lowest.

Regarding the CVI, 99% of the students considered the items appropriate. Regarding the psychometric properties, Cronbach's alpha was 0.90 for all items of the translated and adapted version.

**Figure 2:**
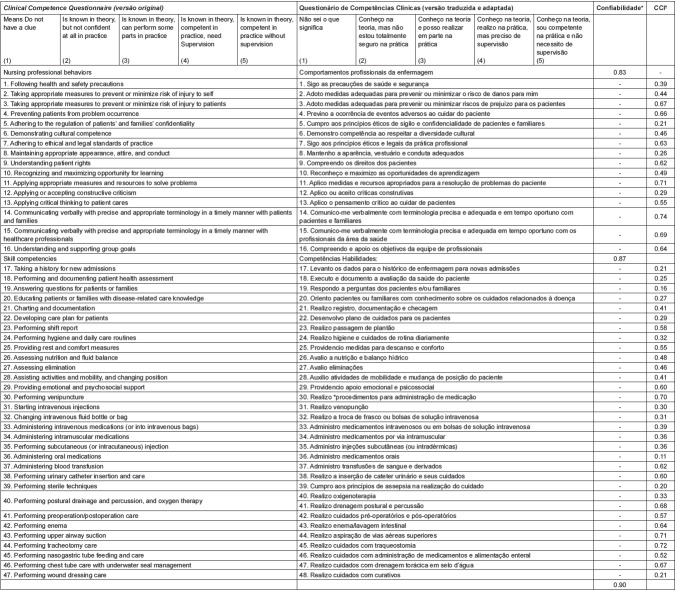
Original version and adapted version of CCQ items. Curitiba, PR, Brazil, 2016

Figure 2 shows the original CCQ version beside the translated and transculturally adapted version to Brazilian nursing graduate students, and their psychometric properties. 

* Reliability = Cronbach's alpha † ICC = Intraclass Correlation Coefficient.

## Discussion

The analysis of items 1 to 13 of the Nursing Professional Behaviors axis showed an important relationship between this axis and the "decision-making" competence of the National Curricular Guidelines of Nursing Undergraduate Courses (*DCENF*).^10^ ^)^ This axis refers to the safe care referred to in the original instrument, which suggests that professionals' performance must be anchored in their ability to make decisions that lead to effectiveness and adequate cost-effectiveness of the workforce related to equipment, medicines, procedures and practices^10^.

Decision-making by nurses is a complex, dynamic and inseparable process of their training; its commitment is considering factors that, when combined, potentiate the quality of the decision and can affect the patient's prognosis and safety; these being: psychological, cognitive, and analytical aspects, information, situations and intuition^11^.

Another aspect to be considered is that nursing constitutes as the largest health workforce in Brazil, concentrating an estimated 1,500,000 professionals. This expressive number indicates how much a direct relation of the category is necessary for patient safety strategies and error prevention^12^.

In order to prevent errors, the ability of judgment and decision making have been the subject of theoretical-practical continuing development in nursing courses, given the need to train nurses who are capable of clinical judgment and evidence-based interventions, thus contributing to reducing adverse events and ensuring the quality of health systems, as well as promoting benefits to patients through safe care^13^.

Items 5, 14 and 15 of the Nursing professional behaviors axis relate to the "communication", referring to the third general competence in the *DCENF*, and which seek to ensure that professionals are accessible and maintain confidentiality of the information accredited to them; a relationship that is also present on item 5 of the instrument. On the other hand, communication skill (according to the *DCENF*) is related to writing and reading skills, which are related to items 18, 19, 20 and 21 of the instrument.

An analysis of the technical skills listed in items 22 to 48 of the original version of the instrument indicate that they are related to the current specific skills and abilities listed by the *DCENF*, which include technical-scientific and ethical-political competencies, and enable nurses to carry out prevention, promotion, protection and rehabilitation in health. Moreover, they are related to general/overall competency of the *DCENF*, which is "healthcare"^10^.

General competence of healthcare points to the training of critical and reflexive professionals engaged in seeking and solving health problems of the individual, the family and the community, respecting ethical and bioethical principles^10^. In this care, clinical reasoning helps the professional to recognize patient priorities and to select the relevant care. We believe that nurses use problem-solving skills in their professional practice on a daily basis, and that these assist them in interventions in the current health context.

Modification of item 4 was based on the concern of one of the specialists regarding a lack of definition of the word "problems" in the original version, since this term could be related to emotional, administrative or physical problems. At the consensus meeting, the experts decided to consider the coexistence of problem and adverse situations, with these meaning impairment to care. Thus, the most appropriate term for this lack in care quality would be at the moment an adverse event (AE), defined by the World Health Organization as unintentional incidents that result in damage arising from care^14^. 

Regarding item 6 - "Demonstrates cultural competence", it was modified to "by respecting cultural diversity", since the term "cultural diversity" would be more comprehensive and broader in scope for nursing students, who should be motivated to develop their skills related to cultural diversity and to learn that individuals are the same regardless of gender, race, marital status, age, socioeconomic status, sexual orientation, beliefs, values and cognitive development, and therefore should be culturally respected. We believe that the inclusion of self-reflection strategies about one's own knowledge related to beliefs, values, acculturation, marginalization, racism, sexism and homophobia can guide nursing training^15^.

Item 21 was expanded to address the issue of checking, since nursing annotation is an essential requirement of care which guarantees patient care continuity^16^, and one of the important characteristics of the medical record is checking medical and nursing prescriptions.

In this sense, Resolution 429/2012 of the Federal Nursing Council^17^ was approved in seeking a judicious direction on how to document nursing practices, and which provides for registering professional actions in the patient's chart and in other documents, either by electronic or traditional means, thus comprising the work and care management processes needed to ensure continuity and quality of care.

Item 30 of the original version of the questionnaire became item 31 of the adapted version of the instrument, while item 31 of the original version became item 30. This change seeks to support a safe way of administering medications by first checking: the correct patient, correct medication, correct application route, correct dose, correct time, correct annotation/recording on patient's medical record, correct patient guidance, the patient's right to refuse the medication^18^, and then to do venipuncture.

Safety in medication administration is a complex competence, requiring attention by nursing professionals who play an important role in this action^19^ from the medication's preparation until administration at the patient's bed.

Despite item 37 not being significantly altered, it brought forth a relevant question to be considered, considering the recommendation that blood transfusions be performed by a licensed and qualified doctor or nursing professional^20^ ^-^ ^21^. Of the students surveyed, 80% evaluated themselves as having the theoretical knowledge and ability to perform transfusions, but only with supervision. This suggests that a reduced number of opportunities to perform this procedure in clinical steps leads to bedside insecurity.

Item 40 of the original version was broken down into two items. The experts identified a need for this alteration considering that postural drainage and percussion is currently not an exclusive practice of nursing in Brazil, which has led some higher education institutions not including this subject in the curriculum of their courses - a situation experienced during the pre-test. However, there would be a need to standardize the scores if a comparison was carried out between the results of samples using both the original and the translated/adapted instrument.

A study carried out in 2008 in Caxias do Sul, RS, from the analysis of 69 medical records identified that percussion was not present in nurses' records, although the percussion technique of the cardiorespiratory system must necessarily be understood by nursing students in seeking to improve the quality of care provided to the patient, since it is a practice that influences therapeutic behavior^22^.

Regarding the psychometric analysis, the instrument proved to be valid and reliable for the studied sample. It was valid when measuring the clinical competencies of undergraduate students in nursing, which demonstrates that it is adequate for the intended scope; and reliable as it obtained a value of 0.90 for Cronbach's alpha, which is considered a high value, therefore demonstrating that the test items are correlated^7^. Thus, the instrument demonstrated validity and precision with a high degree of internal consistency, a result corroborated by the original instrument^5^.

Regarding the intraclass correlation coefficient, 46 items were classified as very good and reasonable, thus inferring that the instrument has stability ranging from moderate to significant^8^. On the other hand, two items obtained poor correlation, which is justified by the fact that their competencies are related to specific clinical practices, suggesting that these are uncommon during students' practical training.

In turn, content validity obtained 99% agreement between the students and 98% among the experts. Mean scores for each domain found in the Brazilian version were similar to the original study scores of the instrument. Judges' analysis and semantics are part of the construction procedures of measuring an instrument, as well as the initial validity stage^23^; therefore, in complying with this construction step, we assure the scale's content validity.

## Conclusion

The CCQ was translated and transculturally adapted into the Portuguese language and some of its psychometric properties were tested. However, it is necessary to finalize the validation process through further appropriate sampling and statistical tests in order for it to be used by graduating undergraduates in nursing.

The availability of this questionnaire may allow for the self-assessment of student's clinical competence, which is considered to be an essential component of nursing since it assists in obtaining new knowledge, better learning and safer patient care.

We can also consider that the questionnaire may provide teachers and supervisors of nursing practices with parameters regarding the clinical competence progression of future nurses, pointing out what can be developed in the clinical steps. This is a time when students can demonstrate their knowledge, skills and attitudes, considering that the instrument evaluates both behaviors and skills. In health institutions, the QCC could be a useful and viable tool for nursing managers to evaluate novice and beginner nurses. 

## References

[1] Cant R, Mckenna L, Cooper S (2013). Assessing preregistration nursing students'clinical competence: a systematic review of objective measures. Int J Nurs Pract.

[2] Holland A, Smith F, McCrossan G, Adamson E, Watt S, Penny K (2013). Online video in clinical skills education of oral medication administration for undergraduate student nurses: a mixed methods, prospective cohort study. Nurse Educ Today.

[3] Levett-Jones T, Gersbach J, Arthur C, Roche J (2011). Implementing a clinical competency assessment model that promotes critical reflection and ensures nursing graduates readiness for professional practice. Nurse Educ Pract.

[4] West C, Usher K, Delaney LJ (2012). Unfolding case studies in pre-registration nursing education: lessons learned. Nurse Educ Today.

[5] Liou SR, Cheng CY (2014). Developing and validating the Clinical Competence Questionnaire: A self-assessment instrument for upcoming baccalaureate nursing graduates. J Nurs Educ Pract..

[6] Beaton DE, Bombardier C, Guillemin F, Ferraz MB (2000). Guidelines for the Process of Cross-Cultural Adaptation of Self-Report Measures. Spine.

[7] Tavakol M, Dennick R (2011). Making sense of Cronbach's alpha. Int J Med Educ.

[8] Weir JP (2005). Quantifying test-retest reliability using the intraclass correlation coefficient and the SEM. J Strength Cond Res.

[9] Coluci MZ, Alexandre ONMC, Milani D (2015). Construção de instrumentos de medida na área da saúde. Ciênc Saúde Coletiva.

[10] Conselho Nacional de Educação (2001). Câmara de Educação Superior. Resolução CNE/CES Nº 3, de 7 de novembro de 2001. Institui Diretrizes Curriculares Nacionais do Curso de Graduação em Enfermagem.

[11] Johansen ML (2016). Decision Making in Nursing Practice: A Concept Analysis. Nurs Forum.

[12] Duarte SCM, Stipp MAC, Silva MM, Oliveira FT (2015). Eventos adversos e segurança na assistência de enfermagem. Rev Bras Enferm.

[13] Thompson C, Aitken L, Doran D, Dowding D (2013). An agenda for clinical decision making and judgement in nursing research and education. Int J Nurs. Stud.

[14] World Health Organization (2009). WHO Guidelines for Safe Surgery 2009.

[15] Bednarz H, Schim S, Doorenbos A (2010). Cultural Diversity in Nursing Education: Perils, Pitfalls, and Pearls. J Nurs Educ.

[16] Claudino HG, Gouveia EML, Santos SR, Lopes MEL (2013). Auditoria em registros de enfermagem: revisão integrativa da literatura. Rev Enferm UERJ..

[17] Resolução do Conselho Federal de Enfermagem n. 429 de 30 de maio de 2012 (2012). Dispõe sobre o registro das ações profissionais no prontuário do paciente, e em outros documentos próprios da enfermagem, independente do meio de suporte - tradicional ou eletrônico. http://zip.net/bmtnhd.

[18] Elliot M, Liu Y (2010). The nine rights of medication administration: an overview. Br J Nurs.

[19] Abreu C da CF, Rodrigues MA, Paixão MPBA (2013). Erros de medicação reportados pelos enfermeiros da prática clínica. Rev Enferm Ref.

[20] Portaria n. 2712 de 12 de novembro de 2013 (BR). (2010). Redefine o regulamento técnico de procedimentos hemoterápicos. http://zip.net/bftnfl.

[21] Resolução do Conselho Federal de Enfermagem n. 306 de 25 de abril de 2006 (BR). (2006). Normatiza a atuação do Enfermeiro em Hemoterapia.

[22] Patrício ACF de A, Alves K de L, Santos J de S, Araruna P da C, Duarte MCS, Rodrigues MMD (2015). Exame físico cardiorrespiratório: conhecimento de estudantes de enfermagem. Rev Pesqui Cuid Fundam.

[23] Lobão WM, Menezes IG (2012). Construction and content validation of the scale of predisposition to the occurrence of adverse events. Rev. Latino-Am. Enfermagem.

